# Relationship between mean platelet volume and metabolic syndrome in Chinese patients

**DOI:** 10.1038/s41598-018-32751-1

**Published:** 2018-10-01

**Authors:** Fengxiao Zhao, Ziyu Yan, Zhaowei Meng, Xue Li, Ming Liu, Xiaojun Ren, Mei Zhu, Qing He, Qing Zhang, Kun Song, Qiyu Jia, Chunmei Zhang, Huiying Wang, Xiaoxia Liu, Xuemei Zhang, Xiaoran Wang, Zhengzhou Pan, Xiangxiang Liu, Wan Zhang

**Affiliations:** 1 Department of Nuclear Medicine, Tianjin Medical University General Hospital, Tianjin, P. R. China; 2 Department of Endocrinology and Metabolism, Tianjin Medical University General Hospital, Tianjin, P. R. China; 3 Department of Health Management, Tianjin Medical University General Hospital, Tianjin, P. R. China

## Abstract

Mean platelet volume (MPV) is a determinant of activation and variability of platelets (PLT). The focus of this study was to to investigate MPV values in patients with and without metabolic syndrome (MS). It also evaluates the association between them. There are close connections among MPV, MS, and cardiometabolic risk. We compiled age, body mass index, blood cell counts, MPV, and other data of 59976 self-reported healthy volunteers (28428 male, 31548 female), 24.65% of who have MS. The mean age of the group was 48.21 years old. The data was grouped by sex and values of data between men and women groups were analyzed by independent sample’s t-test. The relationship between sex and MS was evaluated by chi-square tests. Crude odd ratios of MS between MPV quartiles and 95% confidence intervals were analyzed by binary logistic regression in this study. We found women had higher levels of MPV (10.09 vs. 9.98, P < 0.01) and PLT (228.68 vs. 212.11, P < 0.01) than men. In females, the prevalence of MS was higher in low MPV group than in high MPV groups. The odds of having MS were significantly lower in higher MPV quartiles compared with MPV Quartile 1 in women (Adjusted OR < 1, P < 0.01). This study indicated that MS was inversely associated with MPV in females only.

## Introduction

Metabolic syndrome (MS) means a cluster of cardiometabolic risk factors including elevated blood pressure, central obesity, atherogenic dyslipidemia and impaired glucose metabolism^1,2^. A study reported that 20% of adults in the Western world have MS^3^. Getting complete blood count (CBC) is a convenient and affordable way to reveal the hematological status and, can probably provide important information indicating MS. Some studies have demonstrated that the levels of hemoglobin, red blood cell (RBC), white blood cell (WBC), and blood platelet (PLT) were higher in adults with MS^4^. Other studies have found a positive correlation between PLT aggregation function and mean platelet volume (MPV)^5,6^. Larger and more reactive PLTs increase the possibility of thrombosis. MPV is associated with a wide variety of diseases such as cardiovascular disease (CVD) and diabetes mellitus (DM)^7^. Furthermore, some investigations found MS could predispose people to the development of CVD and DM. These investigations would suggest a high level of MPV as a potential risk factor for MS. But, there are controversies about the associations between MPV and MS. For instance, Aypak *et al*.^8^ showed that patients with MS had a significantly lower MPV level and this, phenomenon only appeared in females. And the volunteers of this investigation were only pre-pubertal children. However, Lee *et al*.^9^ found that MPV in patients with MS was significantly higher compared with the control groups. In addition, gender differences have not been given enough attention through available literature. Therefore, here we aimed to research the association between MPV and MS in different sex group.

## Results

### Characteristics of the participants in different genders

Table 1 showed significant data differences in different genders. Males were older than females. Body mass index (BMI), systolic blood pressure (SBP), diastolic blood pressure (DBP), alanine aminotransferase (ALT), creatinine (Cr), triglycerides (TG), glucose (GLU), WBC, RBC and platelet distribution width (PDW) were significantly higher in males than in females. Total cholesterol (TC), high density lipoprotein (HDL), MPV and PLT were significantly lower in males than in females.

**Table 1:** Population characteristics based on different genders.

	Total	Male	Female	T value
Case number	59976	28428	31548	
Age (years)	48.21 ± 12.87	49.08 ± 12.61	47.43 ± 13.03	15.711**
Weight(kg)	67.99 ± 12.30	75.69 ± 10.71	61.05 ± 9.08	181.087**
BMI (kg/m^2^)	24.75 ± 3.44	25.64 ± 3.18	23.95 ± 3.47	61.824**
Waist(cm)	83.71 ± 10.59	89.11 ± 8.89	78.84 ± 9.60	135.707**
SBP (mmHg)	123.06 ± 17.62	125.35 ± 16.72	120.99 ± 18.16	30.546**
DBP (mmHg)	77.35 ± 11.05	80.35 ± 11.13	74.65 ± 10.24	65.336**
ALT (U/L)	21.47 ± 12.53	24.92 ± 13.52	18.36 ± 10.64	66.428**
Cr (μmol/L)	68.81 ± 14.22	78.82 ± 11.59	59.78 ± 9.56	220.202**
TC(mmol/L)	5.15 ± 0.98	5.08 ± 0.92	5.21 ± 1.03	−16.350**
TG(mmol/L)	1.42 ± 0.84	1.61 ± 0.91	1.24 ± 0.72	55.660**
HDL (mmol/L)	1.45 ± 0.36	1.31 ± 0.32	1.57 ± 0.36	−91.207**
GLU(mmol/L)	5.13 ± 0.82	5.25 ± 0.89	5.02 ± 0.73	35.914**
WBC(×10^9^/L)	5.53 ± 1.16	5.71 ± 1.18	5.37 ± 1.11	36.437**
RBC(×10^12^/L)	4.60 ± 0.31	4.79 ± 0.24	4.43 ± 0.28	169.042**
PDW(fL)	17.37 ± 19.12	17.43 ± 19.54	17.31 ± 18.74	0.776**
MPV(fL)	10.04 ± 1.10	9.98 ± 1.09	10.09 ± 1.11	−12.517*
PLT(×10^9^/L)	220.83 ± 45.74	212.11 ± 43.59	228.68 ± 46.21	−45.057**

BMI = body mass index, SBP = systolic blood pressure, DBP = diastolic blood pressure, ALT = alanine aminotransferase, Cr = creatinine, TC = total cholesterol, TG = triglycerides, HDL = high-density lipoprotein cholesterol, GLU = glucose, WBC = leukocyte, RBC = erythrocyte, PDW = platelet distribution width, MPV = mean platelet volume, PLT = platelet.

*P < 0.05, **P < 0.01 (analyzed by independent sample’s t test).

### Incidence of MS in different genders by MPV quartiles

Date was grouped by MPV quartiles, and within each MPV quartile MS incidences for females and males were calculated and compared. The same analysis was also done for the overall group. Females with lower MPV were more likely to be afflicted with MS. Males showed significantly higher overall incidence of MS than females (Table 2).

**Table 2:** Incidence of metabolic syndrome in different genders.

	Incidence (and case number count) in different MPV quartiles				
	Quartile 1	Quartile 2	Quartile 3	Quartile 4	Total
Male					
Normal^#^	70.01% (5640)	70.93% (4527)	70.84% (5189)	71.00% (4732)	70.66% (20088)
Metabolic syndrome^#^	29.99% (2416)	29.07% (1855)	29.16% (2136)	29.00% (1933)	29.34% (8340)
Female					
Normal^#^	77.21% (6883)	79.11% (6563)	80.45% (5303)	82.01% (6352)	79.56% (25101)
Metabolic syndrome^#^	22.79% (2032)	20.89% (1733)	19.55% (1289)	17.99% (1393)	20.44% (6447)
Chi-square value^**^**^					
Total^^^	113.339**	130.571**	172.573**	244.878**	637.872**

MPV = mean platelet volume.

^#^Metabolic syndrome was diagnosed according to the National Cholesterol Education Program Adult Treatment Panel III criteria 3 (NCEP ATP 3).

^^^Comparing the incidence of normal and/or Metabolic Syndrome in different genders by Chi-square test.

*P < 0.05, **P < 0.01.

### Correlations between MPV and other key variables

MPV displayed significant negative relationships with age, SBP, DBP, Cr, TC, GLU and PLT in both genders. But MPV demonstrated positive relationships with ALT, TG, WBC and RBC in men, as well as ALT and HDL in women (Table 3).

**Table 3:** Pearson bivariate correlation coefficients.

	MPV in male	MPV in female
Age	−0.057**	−0.022**
Weight	0.034**	−0.010
BMI	0.026**	−0.011
Waist	0.017**	−0.014**
SBP	−0.028**	−0.043**
DBP	−0.025**	−0.034**
ALT	0.062**	0.016**
Cr	−0.005**	−0.019**
TC	−0.046**	−0.071**
TG	0.022**	−0.041**
HDL	0.006	0.017**
GLU	−0.051**	−0.085**
WBC	0.020**	−0.010
RBC	0.058**	−0.003
PLT	−0.388**	−0.413**

BMI = body mass index, SBP = systolic blood pressure, DBP = diastolic blood pressure, ALT = alanine aminotransferase, Cr = creatinine, TC = total cholesterol, TG = triglycerides, HDL = high-density lipoprotein cholesterol, GLU = glucose, WBC = leukocyte, RBC = erythrocyte, MPV = mean platelet volume, PLT = platelet.

*P < 0.05, **P < 0.01.

### Risks of MS in different MPV quartiles

Binary logistic regression models were conducted to calculate the ORs of MS among MPV quartiles using the lowest MPV quartile as reference for both genders (Table 4). For women, significant risks of MS were demonstrated in low MPV quartiles, with or without adjustment for age and BMI. However, MPV was not a significant indicator in men.

**Table 4:** The risks of MS according to MPV quartiles.

	Male			Female		
	Parameter values	Crude OR (CI)^^^	Adjusted OR (CI)^$^	Parameter values	Crude OR (CI)^^^	Adjusted OR (CI)^$^
MPV Quartile 1	MPV ≤ 9.30(reference)			MPV ≤ 9.40(reference)		
MPV Quartile 2	9.30 < MPV ≤ 9.800	0.957 (0.890–1.028)	0.966 (0.890–1.048)	9.40 < MPV ≤ 10.0	0.894** (0.832–0.962)	0.889** (0.817–0.969)
MPV Quartile 3	9.80 < MPV ≤ 10.50	0.961 (0.897–1.030)	0.929 (0.858–1.005)	10.0 < MPV ≤ 10.60	0.823** (0.761–0.891)	0.815** (0.744–0.894)
MPV Quartile 4	MPV > 10.50	0.954 (0.888–1.024)	0.916 (0.844–0.993)	MPV > 10.60	0.743** (0.688–0.802)	0.717** (0.655–0.784)

MPV = mean platelet volume, OR = odds ratio, CI = confidence interval.

^#^Metabolic Syndrome was diagnosed according to the National Cholesterol Education Program Adult Treatment Panel III criteria 3 (NCEP ATP 3).

^^^Logistic regression model with quartile 1 as reference, including no covariates.

^$^Logistic regression model with quartile 1 as reference, including age, BMI as covariates.

*P < 0.05, **P < 0.01.

## Discussion

The association between MPV and MS has been reported with controversies^8–14^. In the current study, we demonstrated that MPV was inversely related with MS in women, which was in agreement with several previous reports. Aypak *et al*.^8^ demonstrated that the MPV was positively associated with MS in pre-pubertal girls. He dismissed a previous controversial point that MPV was significantly increased in adults with MS^13^. This discrepancy could be due to unhealthy lifestyles in adults like lack of sleep and consumptions of alcohol and many drugs used from adolescence to adulthood. Aypak *et al*.^8^ argued that it should be worth noting that drugs, such as clopidogrel, statins and angiotensin-converting enzyme inhibitors which could influence MPV levels, were often used in adults with MS^15–17^. Park *et al*.^14^ also proved that MPV was negatively correlated with MS, but only in women. He thought PLT counts and MPV were influenced by different factors such as race, smoking habits, gender, physical activity and alcohol consumption. In addition, men tend to have a higher rate of smoking than women. Park *et al*.^14^ argued that in contrast to men with much confounding factors, women, especially pre-pubertal girls were the better group for testifying the association between MS and MPV, and the results should be more reliable.

The underlying reason of the relationship among MPV, PLT and MS deserves discussion. It has been suggested that the MPV directly associated with PLT aggregation function. Furthermore, PLT aggregation function has been proven to be increased not only both in acute coronary syndrome and in the presence of cardiovascular risk factors such as DM, hypertension and dyslipidemia^5,12,14^. Higher MPV can increase volume of PLTs and enable more active abilities of metabolism and enzymatic reactions. Hence, bigger PLTs have greater prothrombotic potential than smaller ones. There is a negative feedback between MPV and PLT count. While the level of MPV is becoming lower, the PLT aggregation function becomes weaker. So bone marrow cells produce more PLTs to support metabolism and enzymatic reactions in the human body. Therefore, MPV and PLT count are inversely related^4^. The relationship between PLT and MS was shown by Zhou *et al*.^18^, who illustrated that PLT count was a protective factor for MS for men, but a risk factor for women. The mechanism linking PLT and MS could be due to cytokines and insulin resistance, for instance. Jesri *et al*.^13^ demonstrated that the number of MS components was positively associated with PLT count. A feature of MS is increasing adipose tissue, which can secrete different kinds of cytokines and adipokines such as leptin, tumor necrosis factor-a, adiponectin, and interleukin 6. These proinflammatory cytokines provide an environment of chronic low-grade inflammation and thus increase PLT counts^8,14,19^. PLT lifespan is shorter in subjects with insulin resistance, which can make the PLT count increase^20^. Additionally, the interactions between thrombosis and inflammation should provide another potential mechanism linking PLT count and MS^21^. Collectively speaking, a low level of MPV can increase PLT counts, which in turn will eventually lead to the development of MS. Thus, it could be understood in our investigation that the risk of MS was reduced with increasing MPV in Chinese women.

There are some limitations in our investigations. Firstly, it was a cross-sectional design which cannot confirm the casual relationship. Prospective and interventional studies are essential to explain the causality question in the future. Secondly, although we filtered the subjects according to strict exclusion criteria, some volunteers weren’t aware of their healthy condition, which could have led to error in our investigation. Thirdly, we did not measure inflammatory cytokines such as interleukin and tumor necrosis factor α in this project because of a budget shortage. Similarly, insulin resistance was not measured in this study due to a budget shortage as well. Finally, detailed food recall and some other consumed drugs, which could influence hematological parameters or metabolism, should be recorded in specific details for risk stratification in further research.

In conclusion, this research proves that increased MPV is inversely associated with the risk of developing MS in Chinese women, but not in men. A larger scale and prospective study should be performed in the future for further verification and validation.

## Methods

### Design

A cross-sectional, community-based health-check program has been commenced in Tianjin Medical University General Hospital since 2007, with assistance of many departments. Written consents were obtained and the protocol was developed and executed as previously by our group^18,22–29^. Briefly, we asked all of the self-reported healthy participants to fill out a questionnaire. After collecting personal information, blood samples were obtained. In order to avoid influential factors, subjects meeting any of the following criteria were excluded: participants with histories of hepatic, hematological, inflammatory, infectious, renal, cardiovascular, gastro-intestinal, thyroidal, immunological or oncological diseases; participants taking any medicine that might influence WBC, inflammation, tuberculosis; TG higher than 6.0 mmol/L, SBP higher than 180 mmHg, GLU higher than 10.0 mmol/L. A total of 59976 (28428 male, 31548 female) eligible patients were admitted to this research from September 2010 to September 2015.

### Informed consent

All participants in this research provided their written consents.

### Ethics

The institutional review board and ethic committee of Tianjin Medical University General Hospital approved the ethical, methodological and protocol aspects of this investigation. We confirm that all methods in the current study were carried out in accordance with the relevant guidelines and regulations.

### Sample and measurement

When the participants visited our department, anthropometric measurements, as well as fasting blood were tested. Body height (BH) and body weight (BW) were measured without wearing heavy coats and without shoes, and BMI was calculated as weight divided by height squared (kg/m^2^). Peripheral venous blood samples were collected for indicators: GLU, TC, HDL, ALT, Cr, and TG were determined by an automated analyzer (Hitachi Corporation, Tokyo, Japan); PLT, MPV are measured on an automated hemotological analyzer (Sysmex Corporation, Kobe, Japan). We found that sampled platelets swell in an EDTA tube as time increases and consequently MPV values increase. So MPV was analyzed in 1 hour.

### Definition

MS was determined according to the National Cholesterol Education Program Adult Treatment Panel III criteria 3^2^. Thus, MS was defined by the presence of three or more of those criteria: (1) Abdominal obesity (waist circumference >88 cm in women, and >102 cm in men); (2) A high TG level ≥1.7 mmol/L; (3) A low HDL cholesterol level <1.3 mmol/L for women, and <1.0 mmol/L for men; (4) A high blood pressure (systolic, ≥130 mm Hg; and/or diastolic, ≥85 mm Hg); (5) A high GLU concentration ≥5.6 mmol/L.

### Statistical analysis

We used Kolmogorov-Smirnov to assess normality of distribution of continuous variables. All continuous variables were presented as mean ± standard deviation (SD). Differences of the parameters between males or females were evaluated by independent sample’s t test. Then, MPV was divided into quartiles. Prevalence differences were compared by Chi-square test. Pearson bivariate correlation was calculated among MPV and other variables. Binary logistic regression analysis was performed to analyze the odds ratio of MS in different MPV levels, with adjustment. We used automated regression to select variables, which were included in Binary logistic regression. All analyses were conducted using Statistical Product and Service Solutions (SPSS version 17.0, Chicago, IL, USA). Statistical significance was defined as P < 0.05.
